# Performance-Based Seismic Design of Steel Frames Utilizing Colliding Bodies Algorithm

**DOI:** 10.1155/2014/240952

**Published:** 2014-08-14

**Authors:** H. Veladi

**Affiliations:** ^1^Department of Civil Engineering, University of Tabriz, Tabriz, Iran; ^2^The Research Center of Optimization and Engineering, Tabriz, Iran

## Abstract

A pushover analysis method based on semirigid connection concept is developed and the colliding bodies optimization algorithm is employed to find optimum seismic design of frame structures. Two numerical examples from the literature are studied. The results of the new algorithm are compared to the conventional design methods to show the power or weakness of the algorithm.

## 1. Introduction

The traditional seismic design practice entails specifying the desired performance objective, and subsequently the structure is designed to meet specific performance levels. Performance-based design is a more general approach which tries to design buildings with predictable loading-induced performance, rather than being based on prescriptive mostly empirical code specifications. The earthquakes and strong winds are the two major loading conditions imposed on buildings and the performance-based seismic design is becoming well accepted in professional practice for the design of buildings under seismic loading [1]. In performance-based seismic design, the criteria are expressed in terms of achieving a set of performance objectives while the structure is under levels of seismic hazard [2].

Performance-based design methodology allows a significantly different approach for formulating optimization problems, leading to the field of performance-based design optimization (PBDO) [3–7]. In recent years, extensive research has been carried out on RBDO problems [8–12]. Also, various approaches were developed using sequential approximate programming concept [13, 14], the optimality criteria (OC) algorithm [15], and a line search strategy [15]. The metaheuristic algorithms such genetic algorithm (GA) as well as ant colony optimization (ACO) [15], particle swarm optimization (PSO) [16], and charged system search (CSS) [17] (developed by Kaveh and Talatahari [18]) are also some other variants which were utilized for PBDO problems.

This paper presents a new developed method, the so-called colliding bodies optimization algorithm (CBO) [19], to determine optimum design of steel frames considering four performance levels. The nonlinear analysis is required to reach the structural response at various performance levels. Nonlinear static analysis, as a pushover analysis, is a method for performance-based design of structures. Based on this method, earthquake effects such as displacements or forces can be applied to structure statically in some stages from zero to a proposed value, and in each stage the nonlinear internal forces and nodal displacements are calculated and used for the next stage [20]. In [21], a second order analysis of members including geometrical nonlinearly (using semirigid steel framework concept) has been presented to the pushover analysis for performance based seismic design.

## 2. Optimal Performance-Based Seismic Design of Buildings

In structural design, it is desirable to reach a proposed service-ability level with the least usage of the material [16]. Performance level is the required behavior of a structure in different situations. Here, we utilize four performance levels as follows [16].

*(a) Operational Level*. In earthquake situation with probability of exceeding equal to 50% in 50 years structure life, the structure must remain elastic and lateral drift in center of gravity at roof level must be lesser than allowable value:

(1) $$\begin{array}{c} \text{OP}\;\;\text{Level}\;\Delta^{\text{OP}}\leq {\overset{-}{\Delta }}^{\text{OP}}, \end{array}$$

where Δ^OP^ is the lateral drift in center of gravity at roof level and ${\overset{-}{\Delta }}^{\text{OP}}$ is the allowable lateral drift in center of gravity at roof level both in operational level.

*(b) Immediate Occupancy*. In earthquake situation with probability of exceeding equal to 20% in 50 years structure life, lateral drift in center of gravity at roof level must be lesser than allowable value:

(2) $$\begin{array}{c} \text{IO}\;\text{Level}\;\Delta^{\text{IO}}\leq {\overset{-}{\Delta }}^{\text{IO}}, \end{array}$$

where Δ^IO^ is the lateral drift in center of gravity at roof level and ${\overset{-}{\Delta }}^{\text{IO}}$ is the allowable lateral drift in center of gravity at roof level both in immediate occupancy level.

*(c) Life Safety*. In earthquake situation with probability of exceeding equal to 10% in 50 years structure life, lateral drift in center of gravity at roof level must be lesser than allowable value:

(3) $$\begin{array}{c} \text{LS}\;\text{Level}\;\Delta^{\text{LS}}\leq {\overset{-}{\Delta }}^{\text{LS}}, \end{array}$$

where Δ^LS^ is the lateral drift in center of gravity at roof level and ${\overset{-}{\Delta }}^{\text{LS}}$ is the allowable lateral drift in center of gravity at roof level both in life safety level.

*(d) Collapse Prevention*. In earthquake situation with probability of exceeding equal to 2% in 50 years structure life, the structure must remain stable and lateral drift in center of gravity at roof level must be lesser than allowable value:

(4) $$\begin{array}{c} \text{CP}\;\text{Level}\;\Delta^{\text{CP}}\leq {\overset{-}{\Delta }}^{\text{CP}}, \end{array}$$

where Δ^CP^ is the lateral drift in center of gravity at roof level and ${\overset{-}{\Delta }}^{\text{CP}}$ is the allowable lateral drift in center of gravity at roof level both in immediate occupancy level.

Roof drift of 0.4%, 0.7%, 2.5%, and 5% of the height of structure is taken as allowable roof drift for OP, IO, LS, and CP performance levels in design optimization process, respectively [22].

The structural optimization problems can be expressed as minimizing the weight of structures as

(5) $$\begin{array}{c} \text{Minimize:}\;W\left(X\right)=\overset{\text{ne}}{\underset{j=1}{\sum }}\rho \cdot L_{j}\cdot A_{j}, \end{array}$$

where *W*(*X*) is the weight of the structure; *X* is the vector of design variables taken from *W*-shaped sections found in the AISC design manual [23]; ne is the number of members; *ρ* is the material mass density; *L*_*j*_ and *A*_*j*_ are the length and the cross-sectional area of the member *j*, respectively.

To predict the seismic demands on building frameworks, a developed computer-based pushover analysis procedure is utilized. The analysis process is inspired of second-order inelastic analysis of semirigid framed structures where rigidity factor is replaced with plasticity factor in stiffness matrix. The detailed explanations are presented in [15, 16].

## 3. Colliding Bodies Optimization

### 3.1. Laws of the Collision between Two Bodies [19]

In physics, collisions between bodies are governed by laws of momentum and energy. When a collision occurs in an isolated system, the total momentum of the system of objects is conserved.

Provided that there are no net external forces acting upon the objects, the momentum of all objects before the collision equals the momentum of all objects after the collision.

The conservation of the total momentum demands that the total momentum before the collision is the same as the total momentum after the collision and is expressed by the following equation:

(6) $$\begin{array}{c} m_{1}v_{1}+m_{2}v_{2}=m_{1}v_{1}^{\prime }+m_{2}v_{2}^{\prime }. \end{array}$$

Likewise, the conservation of the total kinetic energy is expressed by

(7) $$\begin{array}{c} \frac{1}{2}m_{1}v_{1}^{2}+\frac{1}{2}m_{2}v_{2}^{2}=\frac{1}{2}m_{1}v_{1}^{\prime 2}+\frac{1}{2}m_{2}v_{2}^{\prime 2}+Q, \end{array}$$

where *v*_1_ and *v*_2_ are the initial velocity of the first and second objects before impact, respectively; *v*_1_′*v*_2_′ are the final velocity of the first and second objects after impact; *m*_1_ and *m*_2_ are the mass of the first and second objects; and *Q* is the loss of kinetic energy due to the impact.

The formulas for the velocities after a one-dimensional collision are

(8) $$\begin{array}{c} v_{1}^{\prime }=\frac{\left(m_{1}-\varepsilon m_{2}\right)v_{1}+\left(m_{2}+\varepsilon m_{2}\right)v_{2}}{m_{1}+m_{2}},\\ v_{2}^{\prime }=\frac{\left(m_{2}-\varepsilon m_{1}\right)v_{1}+\left(m_{1}+\varepsilon m_{1}\right)v_{2}}{m_{1}+m_{2}}, \end{array}$$

where *ε* is the coefficient of restitution (COR) of two colliding bodies, defined as the ratio of relative velocity of separation to relative velocity of approach.

### 3.2. Theory

The colliding bodies optimization algorithm is one of the metaheuristic search methods recently developed [19]. The idea of the CBO algorithm is based on observation of a collision between two objects in one dimension, in which one object collides with another object and they move toward minimum energy level. It is a population-based search approach, where each agent is considered as a colliding body with mass. The CBO procedure can briefly be outlined as follows.

 (i) The initial positions of CBs are determined with random initialization of a population of individuals in the search space:
  (9) $$\begin{array}{c} x_{i}^{0}=x_{\min}+\text{rand}\left(x_{\max}-x_{\min}\right), \end{array}$$
  where *x*_*i*_^0^ determines the initial value vector of the *i*th CB. *x*_min⁡_ and *x*_max⁡_ are the minimum and the maximum allowable values vectors of variables; rand is a random number in the interval [0, 1]; *n* is the number of CBs.
 (ii) The magnitude of the body mass for each CB is defined as
  (10) $$\begin{array}{c} m_{k}=\frac{1/f_{k}}{\sum_{i=1}^{n}\left(1/f_{i}\right)}, \end{array}$$
  where *f*_*k*_ represents the objective function value of the agent *k*; *n* is the population size. Obviously a CB with good values exerts a larger mass than bad ones.
 (iii) The arrangement of the CBs objective function values is performed in ascending order. The sorted CBs are equally divided into two groups.
  (a) The lower half of CBs (stationary CBs): these CBs are good agents which are stationary and the velocity of these bodies before collision is zero. Thus,
    (11) $$\begin{array}{c} v_{i}=0\;i=1,\ldots ,\frac{n}{2}. \end{array}$$
  (b) The upper half of CBs (moving CBs): these CBs move toward the lower half. Then, the better and worse CBs, that is, agents with upper fitness value of each group, will collide together. The change of the body position represents the velocity of these bodies before collision as
    (12) $$\begin{array}{c} v_{i}=x_{i}-x_{i-(n/2)}\;i=\frac{n}{2}+1,\ldots ,n, \end{array}$$
    where *v*_*i*_ and *x*_*i*_ are the velocity and position vector of the *i*th CB in this group, respectively; *x*_*i*−(*n*/2)_ is the *i*th CB pair position of *x*_*i*_ in the previous group.
(iv) After the collision, the velocity of bodies in each group is evaluated using (8) and the velocities before collision. The velocity of each moving CB after the collision is
  (13) $$\begin{array}{c} v_{i}^{\prime }=\frac{\left(m_{i}-\varepsilon \cdot m_{i-(n/2)}\right)\cdot v_{i}}{\left(m_{i}+m_{i-(n/2)}\right)},\;i=\frac{n}{2}+1,\ldots ,n, \end{array}$$
  where *v*_*i*_ and *v*_*i*_′ are the velocity of the *i*th moving CB before and after the collision, respectively. Also, the velocity of each stationary CB after the collision is
  (14) $$\begin{array}{c} v_{i}^{\prime }=\frac{\left(m_{i+(n/2)}+\varepsilon \cdot m_{i+(n/2)}\right)\cdot v_{i+(n/2)}}{\left(m_{i}+m_{i+(n/2)}\right)}\;i=1,\ldots ,\frac{n}{2}, \end{array}$$
  where *v*_*i*+(*n*/2)_ and *v*_*i*_′ are the velocity of the *i*th moving CB pair before the collision and the *i*th stationary CB after the collision, respectively; *ε* is the coefficient of restitution (COR) and for most of the real objects, and its value is between 0 and 1. It is defined as the ratio of the separation velocity of two agents after collision to the approach velocity of two agents before collision. In the CBO algorithm, this index is used to control of the exploration and exploitation rate [19]. In this paper, the COR decreases linearly from unit (in starting) to zero (in the end of searching).
(v) New positions of CBs are obtained using the generated velocities after the collision in position of stationary CBs. The new positions of each moving CB are
  (15) $$\begin{array}{c} X_{i}^{\text{new}}=X_{i-(n/2)}+\text{rand}\circ v_{i}^{\prime },\;i=\frac{n}{2}+1,\ldots ,n, \end{array}$$
  where **X**_*i*_^new^ and *v*_*i*_′ are the new position and the velocity after the collision of the *i*th moving CB, respectively; **X**_*i*−(*n*/2)_ is the old position of *i*th stationary CB pair. rand is a random vector uniformly distributed in the range (−1,1) and the sign “∘” denotes an element-by-element multiplication.
 Also, the new position of each stationary CB is obtained as
  (16) $$\begin{array}{c} X_{i}^{\text{new}}=X_{i}+\text{rand}\circ v_{i}^{\prime },\;i=1,\ldots ,\frac{n}{2}, \end{array}$$
  where **X**_*i*_^new^, **X**_*i*_, and *v*_*i*_′ are the new position, old position, and the velocity after the collision of the *i*th stationary CB, respectively.
 (vi) The optimization is repeated until a termination criterion, specified as the maximum number of iteration, is satisfied. It should be noted that a body's status (stationary or moving body) and its numbering are changed in two subsequent iterations.

The main steps of CBO algorithm are as follows.

**Step 1 .**

The initial positions of CBs are determined randomly in the search space.

**Step 2 .**

The magnitude of the body mass for each CB is defined.

**Step 3 .**

The arrangement of the CBs objective function values is performed in ascending order. The sorted CBs are equally divided into two groups:stationary and moving CBs.

**Step 4 .**

After the collision, the velocity of bodies in each group is evaluated and the new positions of CBs are evaluated using the generated velocities after the collision in position of stationary moving CBs.

**Step 5 (termination criterion control).**

Steps 2, 3, and 4 are repeated until a termination criterion is satisfied.

Since the algorithm is a continuous algorithm, for solving the discrete problem like the performance-based design of frames it is necessary to make some modifications. Here, we use a rounding function which changes the continuous value of a result to the nearest discrete value. Although this change is simple and efficient, it may reduce the exploration of the algorithm, [24].

## 4. Design Examples

Two building frameworks are selected for seismic optimum design using the metaheuristic algorithm [15–17]. These frames have previously been used to illustrate the pushover analysis technique by Hasan et al. [21] and Talatahari [16]. For the CBO algorithm, a number of 30 CBs are utilized and the maximum number of iterations is considered as 200.

The expected yield strength of steel material used for column members is *σ*_ye_ = 397 MPa, while *σ*_ye_ = 339 MPa is considered for beam members. The constant gravity load *w* is accounted for a tributary area width of 4.57 m and dead load and live load factors of 1.2 and 1.6, respectively. For each example, 30 independent runs are carried out using the new algorithms are and compared with other algorithms.

### 4.1. Four-Bay Three-Story Steel Frame

**Figure 1:**
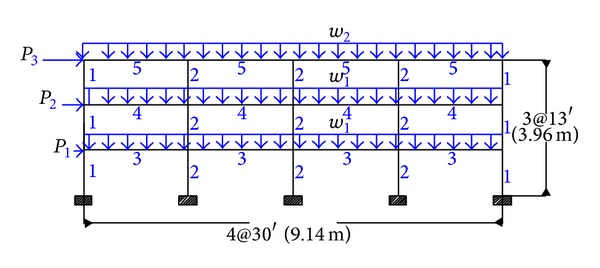
Three-story steel moment frame.

The configuration, grouping of the member,s and applied loads of the four-bay three-story framed structure are shown in Figure 1 [15]. The 27 members of the structure are categorized into five groups, as indicated in the figure. The modulus of elasticity is taken as *E* = 200 GPa. The constant gravity load of *w*_1_ = 32 kN/m is applied to the first and second story beams, while the gravity load of *w*_2_ = 28.7 kN/m is applied to the roof beams. The seismic weight is 4,688 kN for each of the first and second stories and 5,071 kN for the roof story.

**Figure 2:**
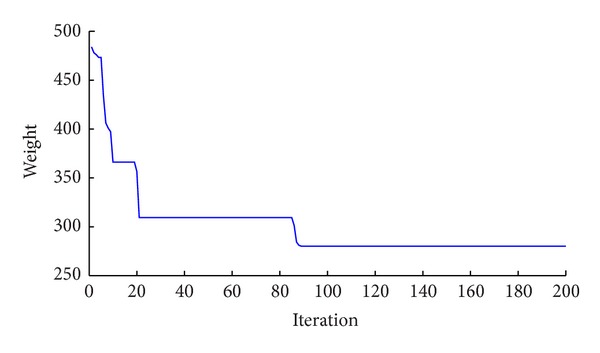
Convergence history of the CBO algorithm for the 4-bay 3-story frame.

The optimum results for the CBO, a hybrid CSS [17], PACO [16], PSO [15], ACO [15], and GA [15] metaheuristic algorithm are presented in Table 1. The new algorithm as well as the CSS and PSACO needs 4500 analyses to reach a convergence while 3900, 6800, and 8500 analyses are required by the ACO, GA, and PSO. After performing optimal design of structure, a pushover analysis is applied to the result obtained using the CBO and roof drifts are controlled in various performance levels. The results show that the roof drifts are less than their corresponding allowable values. The best design for the CBO method has 280.32 kN weight, which is lighter than the conventional design. Although it is heavier than some of metaheuristic algorithms, the differences are small and its standard deviation value in a series of 30 different runs is better than the other methods. The convergence history of the CBO algorithm is presented in Figure 2.

### 4.2. Five-Bay Nine-Story Steel Frame

**Figure 3:**
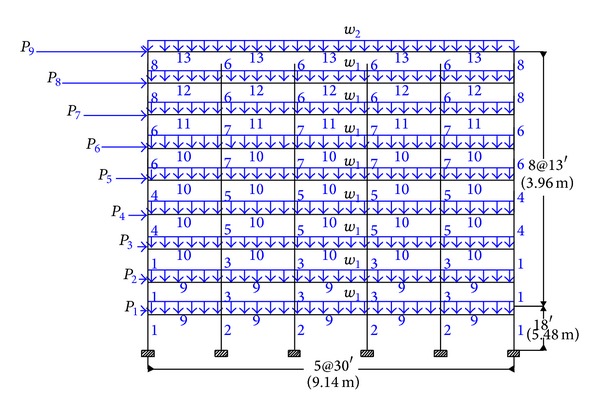
Nine-story steel moment frame.

A five-bay nine-story steel frame is considered as shown in Figure 3. The material has a modulus of elasticity equal to *E* = 200 GPa. The 108 members of the structure are categorized into fifteen groups, as indicated in the figure. The constant gravity load of *w*_1_ = 32 kN/m is applied to the beams in the first to the eighth story, while *w*_2_ = 28.7 kN/m is applied to the roof beams. The seismic weights are 4,942 kN for the first story, 4,857 kN for each of the second to eighth stories, and 5,231 kN for the roof story. In this example, each of the five beam element groups is chosen from all 267 W-shapes, while the eight column element groups are limited to W14 sections (37 W-shapes).

**Figure 4:**
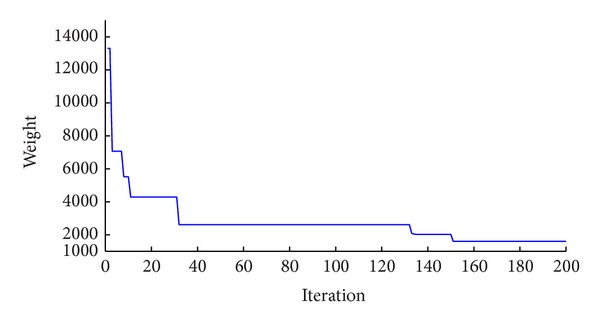
Convergence history of the CBO algorithm for the 4-bay 9-story frame.

The statistical results obtained by the metaheuristic algorithms are presented in Table 2. The best results of the new method is in a frame weighing 1600.25 kN. In order to converge to a solution for the new algorithm, approximately 5,500 frame analyses are required which are less than the 5,600, 6,000, 12,500, and 9,700 analyses necessary for the ACO, PSACO, PSO, and GA, respectively. The hybrid CSS needs only 5,000 analyses to find an optimum result.  Figure 4 shows the convergence history for this example obtained by the CBO algorithm.

## 5. Conclusion Remarks

Performance-based design is a general approach which tries to design buildings with predictable loading-induced performance. In performance-based seismic design, the criteria are expressed in terms of achieving a set of performance objectives while the structure is under levels of seismic hazard. In this paper, the performance-based design of frame structures is formulated to be optimized by the new algorithm, the colliding bodies' optimization. In order to control the lateral drift of building frameworks under seismic loading, a nonlinear analysis is utilized. The analysis method is based on a second order analysis of members including geometrical nonlinearly (using semirigid steel framework concept). The best, average, worst, and standard deviations of minimum weights are obtained by some metaheuristic algorithms as well as the CBO. Although the CBO cannot find the best results, the differences between the results of the CBO algorithm and the best one were small, and the new algorithm did not improve the results significantly.

## Figures and Tables

**Table 1:** The statistical information of performance-based optimum designs for the 4-bay 3-story frame.

Algorithm	This work	Hybrid CSS [17]	PSACO [16]	PSO [16]	ACO [15]	GA [15]	A conventional design [21]
Best weight (kN)	280.32	273.7	279.2	286.3	283.4	303.9	412.9 kN
Average weight (kN)	292.36	286.7	290.4	302.4	294.3	321.5	—
Worst weight (kN)	308.63	297.8	298.5	310.7	303.2	339.7	—
Std. Dev. (kN)	5.786	5.651	6.453	10.453	7.566	14.332	
Average number of analyses	4,500	4,500	4,500	8,500	3,900	6,800	—

**Table 2:** The statistical information of performance-based optimum designs for the 4-bay 9-story frame.

Algorithm	This work	Hybrid CSS [17]	PSACO [16]	PSO [16]	ACO [15]	GA [15]
Best weight (kN)	1600.25	1568.66	1601.32	1682.63	1631.83	1723.1
Average weight (kN)	1660.36	1626.32	1650.55	1725.36	1696.2	1791.4
Worst weight (kN)	1780.62	1725.36	1759.65	1813.25	1786.94	1943.2
Std. Dev. (kN)	31.02	30.35	38.52	66.35	49.33	78.33
Average number of analyses	5,500	5,000	6,000	12,500	5,600	9,700
